# Cytotoxic and Apoptotic Effects of Vanadyl Sulfate on MCF-7 Breast Cancer Cell Line

**DOI:** 10.31661/gmj.v12i.3050

**Published:** 2023-06-21

**Authors:** Maryam Dehdashti, Zahra Abbasy, Hamid Zaferani Arani, Hesam Adin Atashi, Seyed Alireza Salimi-Tabatabaee, Afsaneh Ghasemi, Zhila Fereidouni, Hadi Zare Marzouni, Habib Zakeri, Seyed Abbas Mirmalek

**Affiliations:** ^1^ Young Researchers and Elite Club, Tehran Medical Sciences, Islamic Azad University, Tehran, Iran; ^2^ Faculty of Medicine, Kashan University of Medical Sciences, Kashan, Iran; ^3^ Department of Surgery, Shariati Hospital. Tehran University of Medical Sciences, Tehran, Iran; ^4^ Department of Surgery, Kashan University of Medical Sciences, Kashan, Iran; ^5^ Department of Public Health, School of Health, Fasa University of Medical Sciences, Fasa, Iran; ^6^ Department of Nursing, School of Nursing, Fasa University of Medical Sciences, Fasa, Iran; ^7^ Qaen School of Nursing and Midwifery, Birjand University of Medical Sciences, Birjand, Iran; ^8^ Research Center for Neuromodulation and Pain, NAB Pain Clinic, Shiraz University of Medical Sciences, Shiraz, Iran; ^9^ Department of Surgery, Tehran Medical Sciences, Islamic Azad University, Tehran, Iran

**Keywords:** Vanadyl Sulfate, Breast Cancer, Anti-cancer, MCF-7, Apoptosis, Anti-oxidative

## Abstract

Background: Breast cancer (BC) is the major cause of cancer-related death in women. Some studies have indicated the cytotoxic effects of vanadyl oxide sulfate (VOSO4). This study aimed to evaluate the anti-cancer effect of VOSO4 in the treatment of MCF-7 cell lines.Materials and Methods: The MCF-7 cell line was treated with different concentrations of VOSO4 for 24 and 48 hours. Cell death was measured using the MTT assay. The cell apoptosis rate was measured using Annexin V/Propidium Iodide assay through flow cytometry. Also, the expression levels of p53, P21, Caspase8, superoxide dismutase type 1 (SOD1), Sod2, and Bcl2 mRNAs were assessed, and Western blotting was performed for Sod1 protein.Results: The results showed that the half-maximal inhibitory concentration (IC50) for VOSO4 was 25 and 20 μg/ml for 24 and 48 hours, respectively. Indeed, VOSO4 has dose-dependent cytotoxic effects on the MCF-7. Also, after exposure to VOSO4 for 24 hours, cell apoptosis reached 52% compared with untreated cells. Moreover, after 24 hours of exposure to VOSO4 with IC50 concentration, the expression of p53, P21, Caspase8, Sod1, and Sod2 mRNAs increased (P0.05), and the expression of Bcl2 mRNA was decreased (P0.05). Also, the Western blotting revealed Sod1 protein level markedly increased following exposure to VOSO4 (P0.05). Conclusion: Our results demonstrated that VOSO4 has an apoptotic and cytotoxic effect on BC cells. Therefore, it could be considered a complementary agent for the medical treatment of patients with BC.

## Introduction

Breast cancer (BC) is a multifactorial disease affected by genetic and environmental factors.

It is the most common type of cancer among women after non-melanoma skin cancer [1][2] and the second cause of death from cancer in women after lung cancer [1][2][3]. Currently, treatment options include surgery, radiotherapy, chemotherapy, gene therapy, and so forth. [4][5]. In general, many chemical drugs used in chemotherapy often cause changes in the cell division process, inhibiting the proliferation and differentiation of malignant cells [4][5][6].

Although cytotoxic properties against cancer cells are important in synthesizing these drugs, low side effects on healthy cells are critical issues [4][6].

In recent years, much attention has been paid to the search for new anti-cancer compounds containing metallic ions. Iron was the first metal compound used in drug chemistry [7]. Adopting metal complexes as the drug was developed by applying complexes of platinum, including cisplatin [7][8][9][10][11].

Metal components-binding to the cytotoxic agents-could effectively deliver the medications to the surgery site [8][9]. Indeed, medications with ligands containing manganese, cobalt, and copper have been prepared, binding these complexes to DNA and causing its breakdown [11][12][13].

Evidence reveals that vanadium and its several chemical compounds have important biological activities [12][14][15]. Vanadium at very low concentrations has anti-cancer properties without significant toxicity [8].

Indeed, previous in-vivo and in-vitro studies showed that vanadium compounds have both inhibitory and anti-tumor effects against chemical agent-induced cancers [10][13]. Hence, it may induce cell cycle arrest, DNA fragmentation, and lipoperoxidation of the plasma membrane [11][13]. Anti-cancer effects of vanadium were examined in a study on rat BC models [12].

Subsequent studies have shown the effectiveness of vanadium compounds on various types of human malignancies, including liver, breast, hematopoietic, renal, and epithelial tumors [11][12][13].

Previous studies suggested that different mechanisms for the anti-tumor effects of vanadium through its effect on important cellular processes, such as some metabolic pathways, lead to a reduction or an increase in the expression of various proteins [8][9][11][16][17][18][19]. However, the effects of vanadium on BC and its mechanisms are examined in a few studies.

Therefore, this study aimed to evaluate the anti-tumor effects of vanadyl oxide sulfate (VOSO4) on the MCF-7 cell line.

## Materials and Methods

Cell Culture and Groups

The MCF-7 cell line (Pasteur Institute, Tehran, Iran) was cultured in a complete medium containing high glucose Dulbecco’s Modified Eagle Medium (Gibco, USA) with 10% fetal bovine serum (Gibco, USA) plus 100 mg/ml streptomycin (Gibco, USA) and 100 U/ml penicillin (Gibco, USA). The medium was exchanged two times a week before the addition of fresh media cells and washed with phosphate-buffered saline (Gibco, USA). Also, the MCF-7 cells were divided into the VOSO4 group, which was treated with the half-maximal inhibitory concentration (IC50) dose of VOSO4 (Sigma, USA) for 24 hours and control group (MCF-7 cells without any treatment).

Determining IC50 Dose

The ICD50 dose of VOSO4 was assessed using 3-[4, 5-dimethylthiazol-2-yl]-2, 5 diphenyl tetrazolium bromide (MTT) assay kit (Sigma, USA). In brief, 8000 and 6000 cells were seeded into each well of 96-well plates 16 hours before VOSO4 treatment for 24 and 48 hours, respectively. After 24 and 48 hours, each well was replaced with fresh medium, and MTT powder was added to them according to the manufacturer’s instructions. After four hours, purple formazan sediments appeared at the bottom of each well. These crystals were then dissolved in 200 µl of dimethyl sulfoxide (Sigma, USA), and the absorption of each well was determined by the Biotek ELX800 microplate reader (Bio-Tek, Instruments, Vermont, USA). Also, the Annexin V/PI kit (Roche, Germany) was employed to examine the type of cellular death after treatment as described in the kit manual.

Real-time Polymerase Chain Reaction (PCR)

Total RNA from VOSO4 and control group samples was extracted with TRIzol (Invitrogen, Carlsbad, USA). The same amount of mRNA was used for cDNA synthesis using a cDNA synthesis kit (Takara, Japan). These cDNAs were then used as the template for real-time PCR, which was performed using the Rotor-Gene Q 5plex (Qiagen, Germany) as follows: holding stage (95°C for five minutes), cycling stage (including denaturing step [95°C for 15 seconds], followed by annealing [60°C for 30 seconds], amplification [72°C for 20 seconds, and 40 cycles], and melt curve stage). Primers were designed to specifically amplify cDNA from mRNAs of P53, P21, Caspase8, Bcl2, superoxide dismutase type 1 (SOD1), and Sod2 genes (Table-1).

Western Blot

Cells were lysed, total protein was extracted, and equal amounts of protein from each treated or untreated sample were utilized for SDS-PAGE (10% sodium dodecyl sulfate-polyacrylamide gel electrophoresis). Subsequently, cells were transferred to the nitrocellulose membrane. A 5% non-fat milk incubation for one hour at room temperature was employed for blocking. The membrane was then incubated with the primary antibody (Abcam, UK) against Sod1 protein overnight at 4°C. After that, the membrane was washed three times with a washing buffer containing TBS plus Tween 20 (Gibco, USA). Goat Anti-Rabbit IgG H&L (Abcam, UK) was used as the secondary antibody (mouse monoclonal antibody against glyceraldehyde-3-phosphate dehydrogenase [GAPDH] protein was utilized as the control). The immunocomplexes were visualized with an Immobilon Western Chemiluminescent HRP substrate (Millipore, USA), and appeared bonds were further analyzed by Image J software (version 1.41 National Institutes of Health, Bethesda, USA) for quantification.

Statistical Analysis

All tests were repeated three times. The results were expressed as mean ± standard deviation. Data were analyzed by GraphPad Prism software (version 6.01, GraphPad, La Jolla, CA) using ANOVA and Bonferroni’s tests. The significant difference was set at P<0.05.

## Results

**Table:** Table1. Sequences of Primers and Amplification Reactions Conditions for Evaluation of the Relative Expression

Genes	Primers sequences (5'-3')	Amplicon (bp)
P53	F: GGTACCGTATGAGCCACCTG R: AACCTCAAAGCTGTCCCGTC	166
P21	F: ACTCTCAGGGTCGAAAACGG R: GATGTAGAGCGGGCCTTTGA	150
Bcl2	F: TCTTTGAGTTCGGTGGGGTC R: GTTCCACAAAGGCATCCCAG	153
Sod1	F: ACAAAGATGGTGTGGCCGAT R: AACGACTTCCAGCGTTTCCT	162
Sod2	F: GGTCTGCATTATGCTTGCATGT R: GACTGGAGATACAGGTCTTGGTC	141
Caspase8	F: AGCAGCCTATGCCACCTAGT R: GCTGTAACCTGTCGCCGAG	261
GAPDH	F: AAGTTCAACGGCACAGTCAAGG R: CATACTCAGCACCAGCATCACC	121

**Sod1:** Superoxide dismutase type 1 (SOD1);** GAPDH:**Glyceraldehyde-3-phosphate dehydrogenase

**Figure-1:**
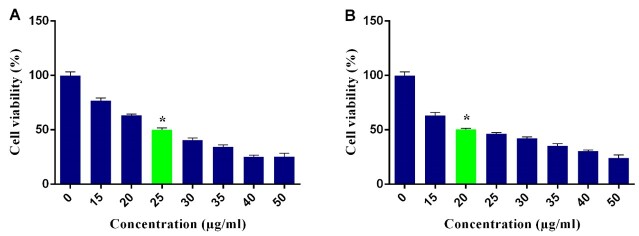
Figure-1. MTT assay results revealed that treatment of MCF-7 cells with 25µg/ml of VOSO4 induces 50% cellular death after 24 hours (A); furthermore, treatment of these cells with 20µg/ml of VOSO4 induces 50% cellular death after 48 hours (B). *P˂0.05 vs. untreated cell

**Figure-2:**
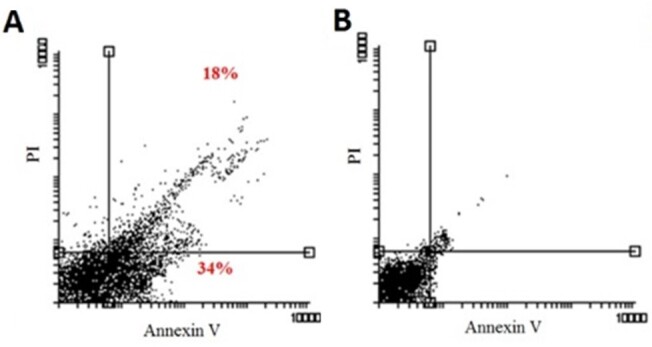
Figure-2 . Annexin V/PI test confirmed that apoptosis was observed in the MCF-7 cells after treatment of these cells with the IC50 dose of VOSO4 for 24 hours. This treatment induces 52% apoptosis (A) in MCF-7 cells compared to the untreated cells as a control (B).

The IC50 Dose of VOSO4

Regarding the MTT assay, 25 and 20 μg/ml of VOSO4 could induce about 50% cellular death after 24 and 48 hours in MCF-7 cells (P˂0.05, Figure-1). Also, Annexin V/PI flow cytometry demonstrated that after treating MCF-7 cells with 25 μg/ml of VOSO4 for 24 hours, 52% of cells underwent apoptosis compared to the control group (P˂0.05, Figure-2).

Treatment with VOSO4 Could Upregulated Apoptotic Genes

**Figure-3:**
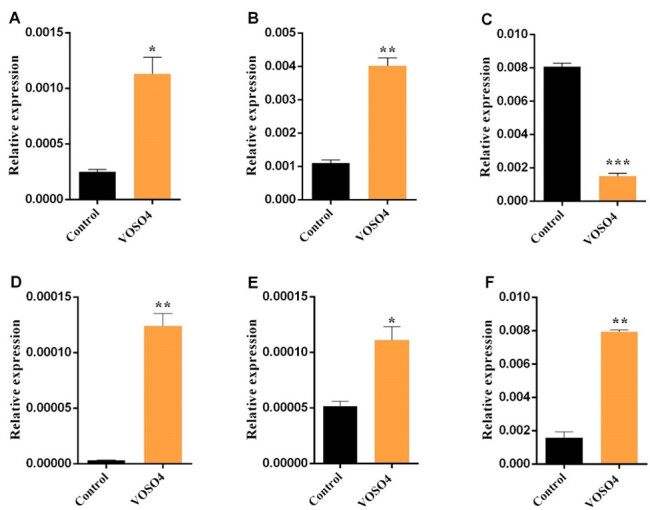
Figure-3. The relative expression of mRNAs for important genes involved in the apoptosis and oxidation pathways. Treatment of MCF-7 cells with the IC50 dose of VOSO4 for 24 hours leads to upregulate the expression of P53 (A), P21 (B), and Caspase8 (C), and downregulates Bcl2 (D), important genes in apoptosis pathway); moreover, this treatment upregulates important genes in oxidation pathway including Sod1 (E) and Sod2 (F). *P˂0.05, **P˂0.01, ***P˂0.001 vs. control

As depicted in Figure-3, the expression level of apoptotic genes, including P53, P21, Caspase8, Sod1, and Sod2 were significantly increased after VOSO4 treatment compared with the control group (P˂0.05). However, the Bcl2 mRNA expression markedly declined in treated cells as an anti-apoptotic gene (Figure-3).

Apoptotic Effect of VOSO4 Could Induced Via Anti-Oxidative Properties

**Figure-4:**
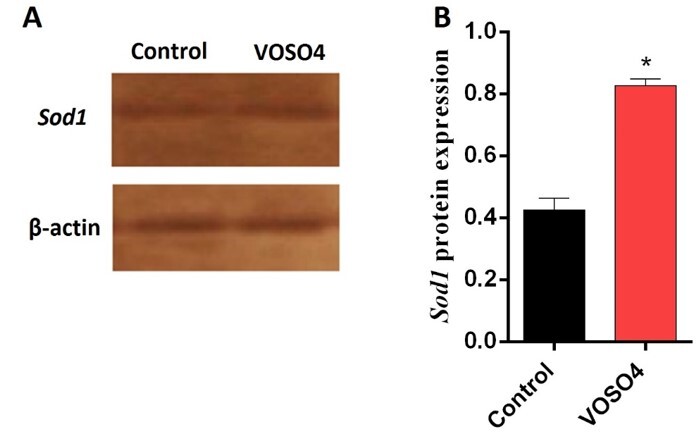
Figure-4. A: Western blot data confirmed that Sod 1 protein level was increased after the treatment of MCF-7 cells with the IC50 dose of VOSO4 for 24 hours. B: Quantification of Western blot data (normalized with β-actin protein) revealed that this treatment increased Sod 1 protein expression level about 2-fold compared with untreated control cells. * P˂0.05 vs. control

Western blot data revealed that the Sod1 protein level was significantly increased after the treatment of MCF-7 cells with 25 μg/ml of VOSO4 for 24 hours (P˂0.05, Figure-4). Indeed, administered IC50 dose VOSO4 for 24 hours could elevate Sod1 level two-fold compared with untreated control cells.

## Discussion

The present study demonstrated that VOSO4 could significantly induce apoptosis in MCF-7 cells via upregulation of apoptotic genes, including P53, P21, and Caspase8, and downregulates Bcl2-important anti-apoptotic gene- as compared to untreated cells. Also, Western blotting analysis revealed overexpression of Sod1, which is the essential protein in the anti-oxidative pathway. Indeed, it seems that VOSO4 could exert its apoptotic effect via the anti-oxidative pathway. Previous studies indicated that the vanadium induced apoptosis in lymphocytes owing to mitochondrial damage and change in the rate of apoptotic proteins (i.e., Bcl2, Bax, and Caspase-3) [20][21]. Vanadium affects various biochemical processes and interacts with many enzymes, including protein kinase, phosphatase, ATPase, peroxidase, ribonuclease, and oxidoreductase [22][23].

A study by Ray et al. [24] indicated that one of the vanadium compounds (NH4VO3) had no significant toxicity on the normal healthy mammary MCF-7 cell line as a control [24]. In addition, Kordowiak et al. showed that the cytotoxic effects of VOSO4 on control cells were significantly less than other vanadium compounds [11]. These findings may indicate that VOSO4 does not have a significant cytotoxicity effect on normal cells. However, its presence in cancer cells could result in the modified expression of p53 and Bax and the regulation of Bcl2 protein as well as anticoagulant activity [25][26][27].

Several studies demonstrated the anti-cancer effects of vanadium in both in-vitro and in-vivo experiments [25][26][28][29]. However, the mechanism of VOSO4 in cancer cell therapy is still not clearly understood. It is argued that this substance in several cellular pathways can affect cell survival and death.

Our results indicated that VOSO4 had significant cytotoxic effects on MCF-7 cancer cells. On the other hand, our results showed an increase in the expression of the genes inducing cell programmed death (p53, p21, Caspase8, Sod1, and Sod2) and a reduction in cell survival (Bcl2) gene.

Das et al. [20] showed that vanadium inhibited the growth of cancer cells. Indeed, the anti-tumor potential of vanadium was reported in the liver, colon, and intestine cancers in-vivo studies and the different cancerous epithelial cells of the in-vitro model [20]. Also, they showed that vanadium could play a major role in moderating the phosphorylation states of various proteins in the cell and affecting many adenosine mono-phosphate cyclic-regulated cellular processes [20].

In a study conducted by Holko et al. [9], the effect of VOSO4 on the growth of human cancer epithelial cells of exocrine tissue was examined. The results showed that VOSO4 significantly inhibited cell growth and reduced carcinoma cells. It also increased the ratio of apoptotic and necrosis cells compared with the control group [9]. However, it should be noted that VOSO4 significantly reduces the vital capability of human cells (BEAS-2B and PNT-2) [9].

Moreover, according to Kordowiak et al. [11], electron microscopic examinations showed that vanadium salts at low concentrations (0.5 μM) destructed cell morphology. Also, higher doses of vanadium salts (more than 2.5-5 μM) damage the cytotoxic state of cellular organelles [11].

In their research, they found that vanadium affects various biochemical processes and can interact with many enzymes [11]. These results and similar studies suggest that VOSO4 induces apoptosis by increasing the expression of genes inducing cell death and preventing cell division by expressing cell survival genes. However, the mechanism of its action on cancer cells is still unknown.

## Conclusion

Our results suggest that VOSO4 was a cytotoxic agent inducing cell death through the expression of apoptosis-inducing genes in MCF-7 cells. It seems that this substance could be considered an appropriate alternative for treating BC with its proper anti-cancer effects.

## Acknowledgments

All authors thank Dr. Mohammad Amin Javidi for providing technical support. Also, we are thankful to the EMA24 English editing service for improving the language of this article.

## Conflict of Interest

All authors declare no conflict of interest.
